# Second Opinions in Internal Medicine

**Published:** 1991-06

**Authors:** John Gavin


### BOOK REVIEWS

### SECOND OPINIONS IN INTERNAL MEDICINE
**Sandler, Fry, Fabb and Godfrey**
Bristol: Clinical Press. Price: £12.50

"Second Opinions in Internal Medicine'' is a short book aimed at junior doctors, general practice trainees and their trainers. It adopts a case study approach to frequent, yet taxing problems that face general practitioners and hospital Consultants. The format adopted is that of GP assessment and management, followed by a referral letter, and then Consultant assessment and reply, finishing with a brief outline of the final outcome. Involvement is encouraged throughout by posing of questions during the analysis of the problem. As reflects many of the problems under discussion, correct answers are not provided.

The format of the book means that it is in danger of being repetitive and the authors have attempted to avoid this by choosing cases that cover a broad spectrum of medicine. In doing so they have succeeded in conveying a considerable amount of information in an easily digestible way. However, that is secondary to the main purpose of the book which is concerned with the relationships and roles of GP and Consultant in shared patient care. In this area the book succeeds in giving a good model of shared care and draws out the different emphasis and contribution of the specialist and the General Practitioner.

Perhaps most importantly at a time when competition is the maxim in medical care, this book succeeds in highlighting the value of integrated health care.

John Gavin

West of England Medical Journal Volume 106)ii) June 1991, *Skrabanek*

